# Clioquinol Inhibits Zinc-Triggered Caspase Activation in the Hippocampal CA1 Region of a Global Ischemic Gerbil Model

**DOI:** 10.1371/journal.pone.0011888

**Published:** 2010-07-29

**Authors:** Tao Wang, Wei Zheng, He Xu, Jia-Min Zhou, Zhan-You Wang

**Affiliations:** Key Laboratory of Medical Cell Biology of Ministry of Education of China, College of Basic Medical Sciences, China Medical University, Shenyang, China; Mental Health Research Institute of Victoria, Australia

## Abstract

**Background:**

Excessive release of chelatable zinc from excitatory synaptic vesicles is involved in the pathogenesis of selective neuronal cell death following transient forebrain ischemia. The present study was designed to examine the neuroprotective effect of a membrane-permeable zinc chelator, clioquinol (CQ), in the CA1 region of the gerbil hippocampus after transient global ischemia.

**Methodology/Principal Findings:**

The common carotid arteries were occluded bilaterally, and CQ (10 mg/kg, i.p.) was injected into gerbils once a day. The zinc chelating effect of CQ was examined with TSQ fluorescence and autometallography. Neuronal death, the expression levels of caspases and apoptosis inducing factor (AIF) were evaluated using TUNEL, *in situ* hybridization and Western blotting, respectively. We were able to show for the first time that CQ treatment attenuates the ischemia-induced zinc accumulation in the CA1 pyramidal neurons, accompanied by less neuronal loss in the CA1 field of the hippocampus after ischemia. Furthermore, the expression levels of caspase-3, -9, and AIF were significantly decreased in the hippocampus of CQ-treated gerbils.

**Conclusions/Significance:**

The present study indicates that the neuroprotective effect of CQ is related to downregulation of zinc-triggered caspase activation in the hippocampal CA1 region of gerbils with global ischemia.

## Introduction

Transient global ischemia, which often occurs during cardiac arrest when the brain is deprived of oxygen and glucose for a short period of time, involves several mechanisms of delayed neuronal cell death, such as excitotoxicity, free radical reaction, mitochondrial dysfunction, inflammation, and neuronal apoptosis [1], [2], [3]. Certain brain regions, especially the hippocampal CA1 field, are selectively vulnerable to transient global ischemia [4], [5], [6]. Interestingly, recent studies suggest that release of synaptic zinc from the zinc-containing excitatory neurons plays a key role in hippocampal neuronal death during cerebral ischemia [7].

In the brain, zinc is mostly bound to proteins, including metalloenzymes and transcription factors, where it plays catalytic or structural roles [8], [9]. Chelatable zinc is located in presynaptic vesicles in a subset of glutamatergic axonal terminals throughout the mammalian forebrain, especially the hippocampus and cortex [10]. Under physiological conditions, synaptically released zinc serves as a neuromodulator and modulates the activity of postsynaptic receptors. In some pathological conditions, such as transient cerebral ischemia, zinc translocation from presynaptic terminals into postsynaptic neuronal cell bodies contributes to the pathogenesis of delayed selective neuronal cell death [11]. Importantly, several studies have shown that chelation of zinc *in vivo* protects against zinc-induced neuronal death in cerebral ischemic animal models. For instance, injection of a zinc chelator, Ca-EDTA, significantly reduces caspase-3 activity and neuronal death resulting from damage caused by global ischemia [11], [12]. Another zinc chelator, DP-b99, has been shown to have neuroprotective properties in animal models as well as stroke patients [13], [14]. These data suggest that zinc chelators are promising agents for neuroprotective therapy after ischemic stroke.

Clioquinol (5-chloro-7-iodo-8-hydroxyquinoline, CQ) is a membrane-permeable and hydrophobic metal chelator [15]. It can selectively bind zinc and copper with greater affinity than calcium and manganese, and can easily cross the blood-brain barrier [15]. Moreover, unlike traditional chelators, CQ is seen as acting to restore biometal homeostasis rather than causing a bulk excretion and is now understood to include a metal chaperone or ionophore activity. CQ could bring metals captured into nearby cells and this activity is most associated with anti-cancer property [16], [17] and decreased interstitial Aβ [18], [19]. Following oral treatment, CQ can significantly reduce the brain β-amyloid burden and can improve the memory impairment of aged AD transgenic mice [15]. In addition, preliminary data from clinical trials have shown that CQ has positive effects on AD patients [20], [21], [22]. Therefore, CQ, with its properties in modulation of cellular biometal metabolism, is being used as a candidate therapeutic strategy for AD [23]. In addition, it is also confirmed that CQ has positive effects in animal models of Parkinson's disease and Huntington's disease [24], [25].

Although the importance of zinc in ischemic neuronal death and the effect of zinc chelation in zinc-induced cell death have been clearly demonstrated, the beneficial effects of CQ have not been investigated in global cerebral ischemia. Therefore, the present study was undertaken to explore the neuroprotective potential of CQ, with special concentration on the inhibition of zinc accumulation in CA1 pyramidal neurons and caspase activity in the hippocampus, in global cerebral ischemia in Mongolian gerbils.

## Results

### Chelating effects of CQ on free zinc ions in the gerbil hippocampus

**Figure 1:**
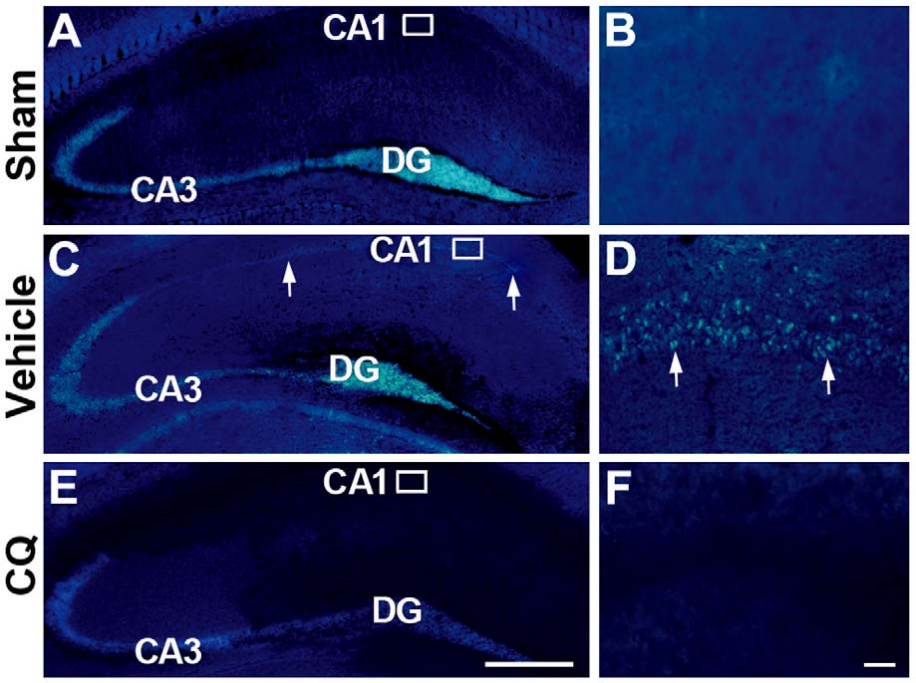
TSQ staining showing the zinc chelating effect of CQ in the gerbil hippocampus. (**A–F**) TSQ fluorescence dye staining in the hippocampus at 3 d after sham-operation (A, B), and in vehicle-treated (C, D), and CQ-treated ischemic gerbils (E, F). Intense zinc fluorescence was observed in the mossy fiber terminals from the DG to the CA3 region. Global ischemia increased the TSQ fluorescence in the CA1 area (C). Striking accumulation of zinc fluorescence was observed in the cell bodies of pyramidal neurons (C, D). Treatment with CQ significantly reduced chelatable zinc in hippocampal mossy fibers and the CA1 region (E, F). Arrows indicate TSQ fluorescence in the pyramidal neuronal cell bodies. CA1: hippocampal CA1 area; CA3: hippocampal CA3 area; DG: dentate gyrus. Scale bars  = 500 µm (A, C, E), 25 µm (B, D, F).

To evaluate the chelatable zinc accumulation and the chelating effects of CQ on zinc ions in the ischemic gerbil hippocampus, we first employed zinc-specific fluorescence indicator dye, N-6-(methoxy-8-quinolyl)-*para*-toluenesulfonamide (TSQ), on hippocampal sections at 3 days after ischemia. In the sham control, intense TSQ fluorescence was found in the mossy fibers from the dentate gyrus (DG) to the CA3 region (Figure 1A), and the CA1 region showed a faint TSQ fluorescence (Figure 1B). In the vehicle-treated ischemic gerbil hoppocampus, clear TSQ fluorescence appeared in the pyramidal neurons of the CA3 region (Figure 1C, D), which is consistent with previous findings that global ischemia leads to a pronounced accumulation of TSQ fluorescence in cell bodies of CA1 pyramidal neurons at 3 days after ischemia [26]. In the CQ-treated ischemic gerbil hippocampus, TSQ fluorescence was markedly reduced not only in the mossy fiber terminals, but also in the cell bodies of CA1 pyramidal neurons (Figure 1E, F).

**Figure 2:**
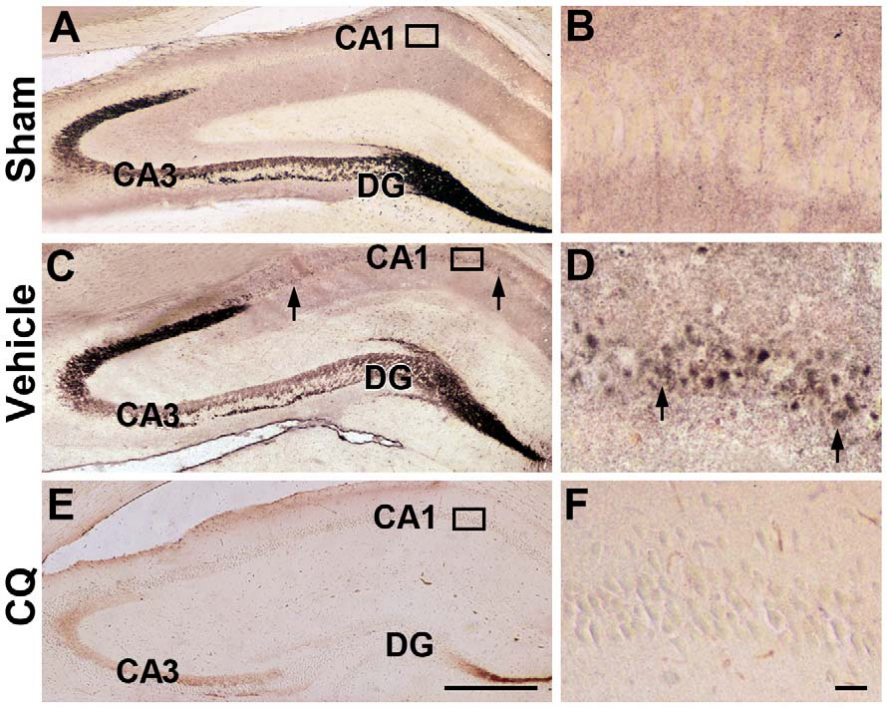
AMG staining showing that CQ injection markedly reduces chelatable zinc in the gerbil hippocampus. (**A–F**) Zinc AMG staining in the hippocampus at 3 d after ischemia. Distinct zinc staining was detected in the hippocampal mossy fibers in sham control (A, B) and vehicle-treated ischemic gerbils (C, D). CQ treatment markedly reduced the zinc staining in the mossy fibers from the DG to the CA3 region (E). Consistent with TSQ fluorescence dye staining, massive accumulation of zinc was observed in the cell bodies of CA1 pyramidal neurons in vehicle-treated ischemic gerbils (D), whereas CQ injection markedly reduced chelatable zinc in CA1 pyramidal neurons (F). Arrows indicate zinc-containing pyramidal neuronal cell bodies. CA1: hippocampal CA1 area; CA3: hippocampal CA3 area; DG: dentate gyrus. Scale bars  = 500 µm (A, C, E), 25 µm (B, D, F).

We also performed immersion autometallography (AMG) staining to further examine the zinc chelating effects of CQ in the ischemic gerbil hippocampus. Since the AMG development procedure is very sensitive to temperature and other parameters, the brain sections from sham control, vehicle-treated and CQ-treated ischemic gerbils were incubated in the AMG developer in the same jar at the same time. Furthermore, sodium diethyldithiocarbamate trihydrate (DEDTC) treatment was carried out, and no AMG-positive staining of brain sections was observed (data not shown), suggesting the specificity of the zinc staining in our immersion AMG staining [27]. In the sham and vehicle-treated gerbils, intense AMG staining was observed in the mossy fibers of the hippocampus (Figure 2A, C). However, the zinc staining in mossy fiber terminals was markedly reduced following CQ treatment (Figure 2E). Importantly, the ischemia-induced zinc accumulation in CA1 pyramidal neurons was very marked in the vehicle-treated ischemic gerbil brain (Figure 2D). In contrast, CQ treatment resulted in virtually no zinc labeling in the cell bodies of CA1 pyramidal neurons (Figure 2F). Consistent with the TSQ fluorescence results, these findings indicate that CQ attenuated the ischemia-induced zinc sequestration in CA1 pyramidal neurons.

### CQ protects against neuronal loss and apoptosis in the hippocampus

**Figure 3:**
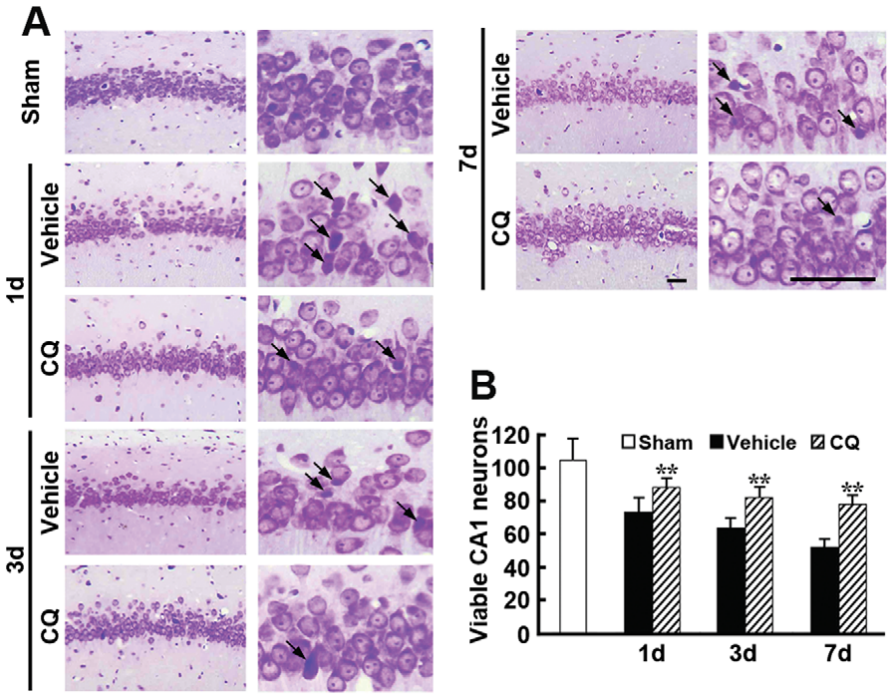
Nissl staining showing the neuroprotective effect of CQ on the pyramidal cells in the gerbil hippocampal CA1 region. (**A**) The number of surviving neurons with round and palely stained nuclei in the CA1 region was increased in CQ-treated gerbils, compared with vehicle-treated controls, at day 1, 3 and 7 post-ischemia. Arrows indicate the dark shrunken damaged neurons. Scale bars  = 25 µm. (**B**) Cell densities were represented on the graphs by counting surviving cells per field under a light microscope, and a significant increase in the numbers of pyramidal neurons in the CA1 were observed in CQ-treated gerbils at day 1, 3 and 7 post-ischemia. ** *p*<0.01 (n = 6).

Global ischemia-induced neuronal loss was assessed by histological examination of Nissl staining. Consistent with previous reports [6], extensive neuronal changes in the CA1 regions of the hippocampus were noted in ischemic gerbil brain. More shrunken neurons with pyknotic nuclei were found in vehicle-treated ischemic gerbils, compared with sham controls (Figure 3A). CQ treatment markedly increased the number of surviving neurons with palely stained nuclei and Nissl substance, compared with vehicle-treated gerbils (Figure 3A). The quantitative results showed that the average numbers of surviving cells in the hippocampal CA1 region of CQ-treated ischemic gerbils were significantly increased compared with those in vehicle-treated controls (*p*<0.01, Figure 3B).

**Figure 4:**
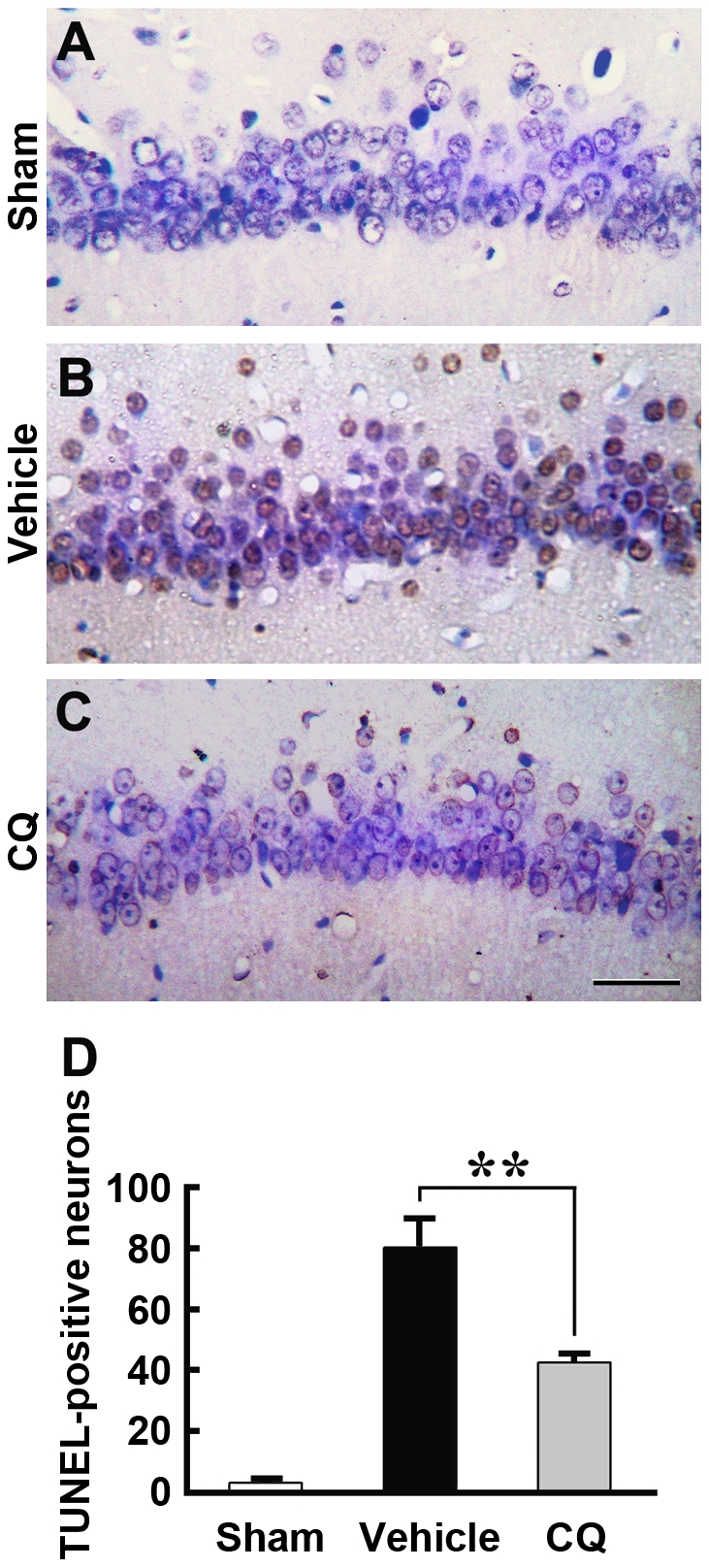
TUNEL staining showing that CQ treatment leads to less apoptotic neurons in the gerbil hippocampal CA1 region. (**A–C**) The TUNEL-positive cells in the hippocampal CA1 region were examined at 3 d after ischemia. Compared with sham controls (A), more TUNEL-positive apoptotic neurons were observed in vehicle-treated ischemic gerbils (B), while the number of TUNEL-positive cells was markedly reduced in ischemic gerbils treated with CQ (C), compared with vehicle-treated gerbils. Scale bar  = 25 µm. (**D**) TUNEL-positive neurons in the CA1 region were counted under a light microscope. Statistically significant reduction in the number of TUNEL-positive apoptotic neurons in the CA1 region were found in CQ-treated gerbils, compared with vehicle-treated controls. ** *p*<0.01 (n = 6).

We further assessed the hippocampal neuronal apoptotic damage by terminal deoxynucleotidyl transferase-mediated dUTP nick end-labeling (TUNEL) staining to determine the changes in the number of neurons showing DNA breaks. As shown in Figure 4, there were very few TUNEL-positive neurons in the CA1 region of the hippocampus of sham control gerbils (Figure 4A). Vehicle-treated ischemic gerbils had many more TUNEL-stained cell nuclei in the CA1 region of the hippocampus (Figure 4B), whereas CQ-treated gerbils had less TUNEL-positive neurons in the hippocampal CA1 region (Figure 4C). Quantitative results revealed that the average numbers of TUNEL-positive nuclei in the hippocampal CA1 region in CQ-treated gerbils were significantly reduced compared with those in vehicle controls (*p*<0.01, Figure 4D). Taken together, both Nissl and TUNEL staining results indicate that treatment with CQ is able to protect against neuronal cell death in the hippocampal CA1 region following an insult produced by transient global ischemia.

### CQ inhibits the activity of caspase-3, caspase-9 and AIF in the hippocampus

**Figure 5:**
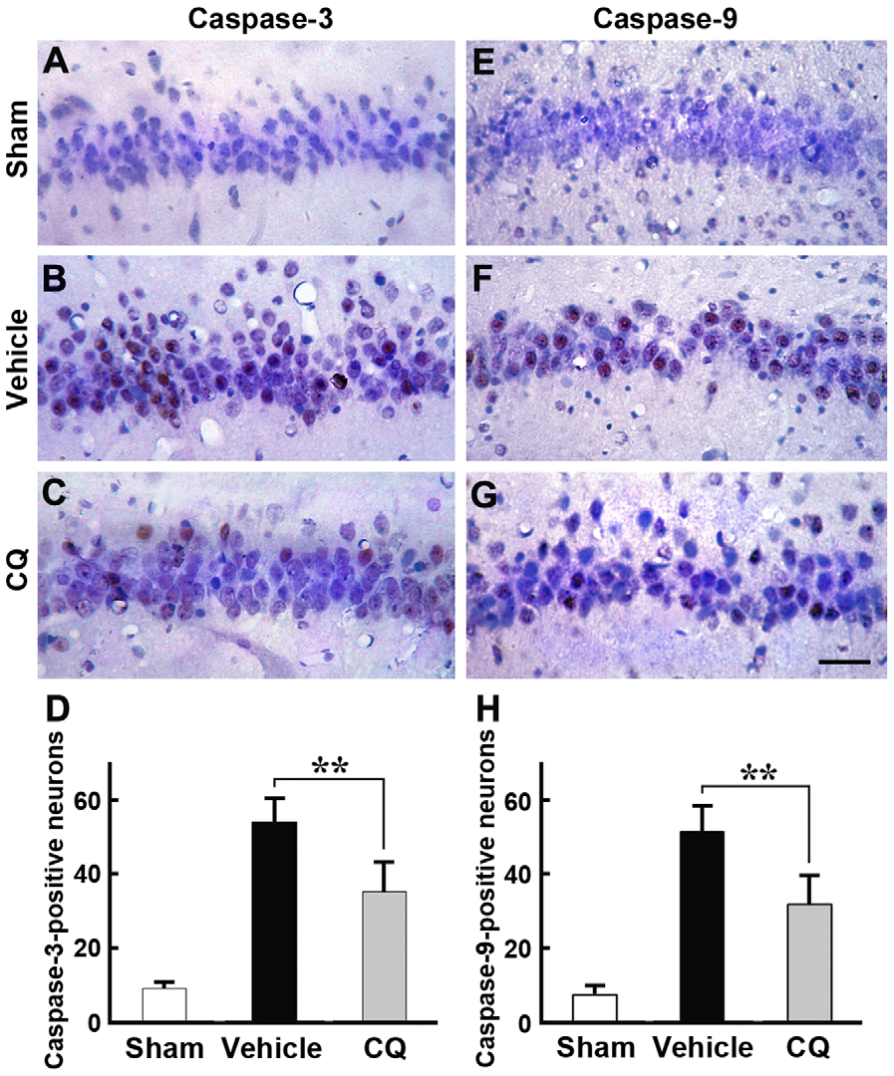
In situ hybridization detection of caspase-3 and -9 in the gerbil hippocampal CA1 region. (**A–G**) The caspase-3- (A–C) and caspase-9-positive neurons (E–G) in the hippocampal CA1 region were analyzed at 3 d after ischemia. The numbers of both caspase-3- and caspase-9-positive neurons were massively increased in vehicle-treated ischemic gerbils (B, F), compared with sham controls (A, E). In the CQ-treated ischemic gerbils, the numbers of caspase-3- (C) and caspase-9-positive cells (G) were markedly reduced compared with vehicle-treated gerbils. Scale bar  = 25 µm. (**D, H**) Statistical analysis showed that the numbers of both caspase-3- and caspase-9-positive neurons were significantly reduced in CQ-treated gerbils, compared with that in vehicle controls. ** *p*<0.01 (n = 6).

To examine whether CQ could modulate the expression of apoptosis-related proteins in the hippocampus, in situ hybridization of caspase-3 and caspase-9 was performed on hippocampal sections from sham control, vehicle-treated and CQ-treated ischemic gerbils (Figure 5). In the CA1 region of the sham control hippocampus, few caspase-3- and caspase-9-positive neurons could be observed (Figure 5A, E). However, in vehicle-treated ischemic gerbils, the number of caspase-3- and caspase-9-stained cells was markedly elevated (Figure 5B, F), whereas CQ treatment significantly reduced the number of caspase-positive neurons (Figure 5C, G). Quantitative results indicated that the number of both caspase-3- and caspase-9-positive cells in CQ-treated ischemic gerbils was significantly reduced in the CA1 region of the hippocampus, compared with that in vehicle-treated ischemic gerbils (*p*<0.01, Figure 5D, H).

**Figure 6:**
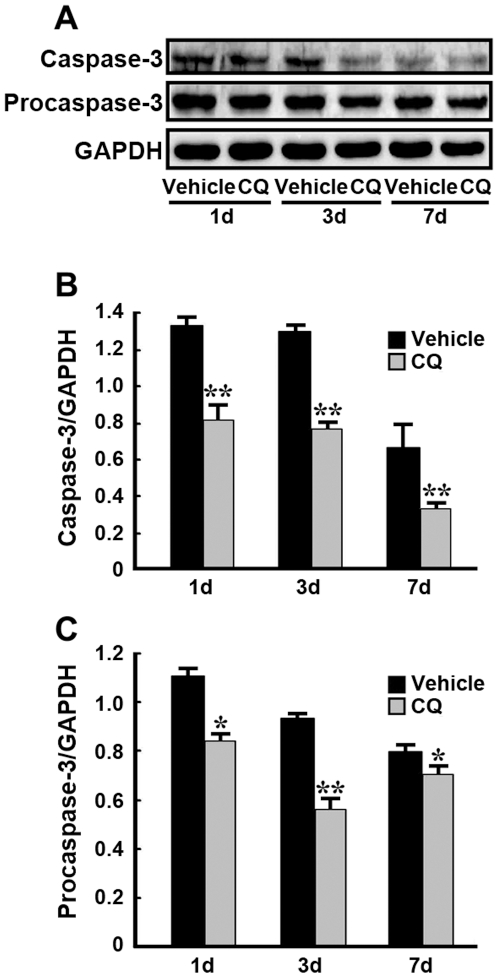
Expression levels of caspase-3 proteins in the gerbil hippocampus. (**A**) Representative images of immunoblots are shown with antibodies against caspase-3 and GAPDH. GAPDH was used as a loading control. (**B, C**) Compared with vehicle-treated ischemic controls, the expression levels of both cleaved caspase-3 (B) and procaspase-3 (C) proteins were reduced significantly in the CQ-treated ischemic gerbil hippocampus at 1, 3, and 7 d after ischemia. * *p*<0.05, ** *p*<0.01 (n = 6).

The expression level of caspase-3 in the hippocampus was further examined using Western blot immunoassay. It is known that caspase-3 is a cytosolic protein and an inactive proenzyme, and is activated by proteolytic cleavage into active subunits. As shown in Figure 6A, the checked proteins were detected in bands located at 32 kDa for procaspase-3 and 20 kDa for cleaved caspase-3 in the gerbil hippocampus. Statistical analyses showed that there were significant reductions in the expression levels of both cleaved caspase-3 (active) and procaspase-3 (inactive) in CQ-treated ischemic hippocampi at 1, 3, and 7d, respectively, compared with vehicle-treated controls (*p*<0.05−0.01, Figure 6B, C).

**Figure 7:**
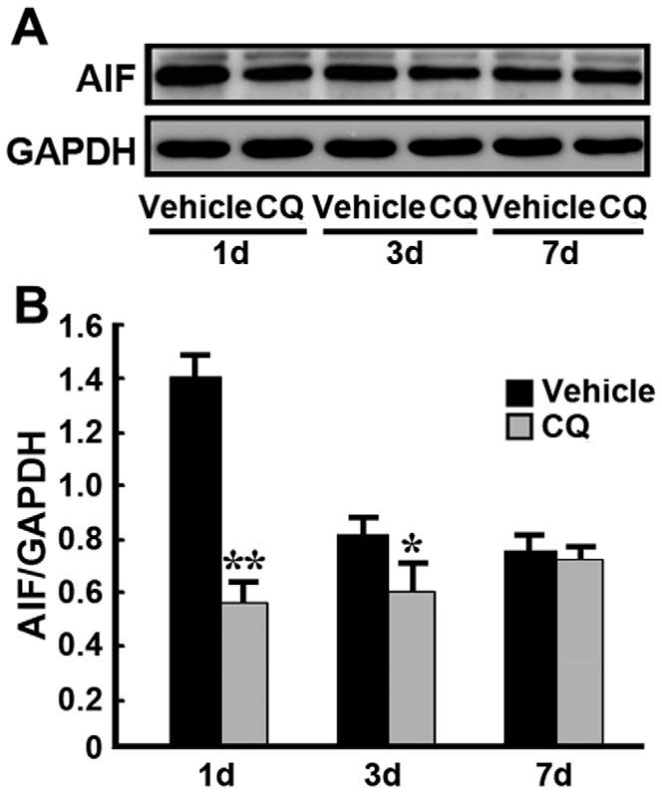
Expression level of AIF protein in the gerbil hippocampus. (**A**) The protein level of AIF in the hippocampus was measured at 1, 3 and 7 d after ischemia. Representative images of immunoblots are shown with antibodies against AIF and GAPDH. GAPDH was used as a loading control. (**B**) The expression level of AIF protein was significantly reduced in the CQ-treated ischemic gerbil hippocampus at 1 and 3 d after ischemia, compared with vehicle-treated ischemic controls. No significant difference in AIF level was detected between vehicle- and CQ-treated ischemic gerbil hippocampus at 7 d after ischemia. * *p*<0.05, ** *p*<0.01 (n = 6).

Apoptosis inducing factor (AIF) is a pro-apoptotic protein associated with caspase-independent neuronal cell apoptosis, and is involved in hippocampal neuronal apoptosis in the ischemic gerbil [28]. Thus, we examined the effect of CQ on the expression of AIF in the hippocampus of the ischemic gerbil using Western blot assay. Our immunoblot results showed that AIF was detected in the band located at 66 kDa (Figure 7A). At 1 and 3d post-ischemia, CQ treatment significantly reduced the level of AIF in the ischemic hippocampus, compared with the vehicle-treated control (*p*<0.05-0.01, Figure 7B). Taken together, these results indicate that the neuroprotective effects of CQ in the hippocampus of the ischemia gerbil may be due to regulating both caspase-dependent and -independent apoptosis signaling pathways.

## Discussion

It is well known that global ischemia causes delayed neuronal death in the selectively vulnerable hippocampal CA1 region and, most interestingly, a delayed zinc rise after ischemic insult but before the onset of cell death is observed in the CA1 neurons [11], [26], [29]. At 3 d after ischemia, the accumulation of zinc is massively increased in CA1 pyramidal neurons [26], indicating that the delayed accumulation of zinc is involved in delayed neuronal death in the gerbil brain. It has been suggested that reduction of zinc accumulation in neurons with a high-affinity zinc chelator, Ca-EDTA, is beneficial for preventing neuronal death after transient cerebral ischemia [11], [26]. In an ischemia rat model, accumulation of zinc was observed specifically in degenerating neurons in the hippocampus, and intraventricular injection of Ca-EDTA could reduce zinc accumulation and prevent hippocampal neurodegeneration [11]. Administration of Ca-EDTA markedly attenuated the late rise in zinc and cell death in the hippocampal CA1 region at 3 days after ischemia in the gerbil [26]. In the present study, we extended our experiments to assess the chelating effects of a zinc chelator, CQ, on the ischemic gerbil hippocampus. Both TSQ fluorescence dye and AMG staining, which are widely employed to demonstrate loosely bound or chelatable zinc ions [30], [31], were employed to assess the effects of CQ on chelatable zinc pools in the gerbil brain. Consistent with a previous report [32], our data showed that CQ reduced the density of TSQ fluorescence and AMG staining in the mossy fibers, suggesting that intraperitoneal treatment of CQ rapidly targets chelatable zinc pools in the hippocampus of the gerbil brain [32]. Most importantly, we showed for the first time that CQ attenuates ischemia-induced zinc accumulation, accompanied by less delayed neuronal death in the CA1 field revealed by Nissl and TUNEL staining. These findings indicate that CQ can target a reduction in delayed zinc accumulation, and is a potential therapeutic approach to preventing neuronal death after transient global ischemia.

The underlying mechanisms whereby zinc is involved in delayed neuronal death and the neuroprotective effects of CQ after ischemic insult are still obscure. In a hippocampal slice model with oxygen-glucose deprivation, zinc rapidly enters neurons, accumulates in mitochondria, and contributes to consequent mitochondrial dysfunction and cell death [33], [34], suggesting caspase-dependent neuronal apoptosis after a toxic zinc insult. In the present study, we measured the expression levels of caspase-3 and caspase-9, two key molecules in the caspase-dependent apoptotic cascade, in the ischemic gerbil hippocampus. Our results showed that ischemic insult markedly increased the number of both caspase-3- and caspase-9-positive neurons in the hippocampal CA1 region 3 days after global ischemia. Together with our TSQ and AMG data and previous reports that increased zinc accumulation in the CA1 neurons was observed after transient cerebral ischemia [26], it is reasonable to speculate that the ischemia-induced zinc rise may trigger the activity of caspases in the CA1 neurons and, consequently, lead to neuronal death. In addition, our data also showed that transient ischemia resulted in an increased level of AIF, a pro-apoptotic mitochondrial molecule and the key factor in the caspase-independent apoptosis signaling pathway [35]. This indicates that ischemia-induced zinc accumulation plays a role in modulating caspase-independent neuronal death following transient cerebral ischemia. In the present study, we further assessed the effects of CQ on modulating the caspase-dependent and -independent death pathways in the hippocampus of the ischemic gerbil. Our data showed that administration of CQ significantly reduced the expression levels of caspase-3, -9, as well as AIF in the hippocampus, 3 days after ischemia in the gerbil. These findings indicate that the neuroprotective effect of CQ involves regulation of caspase-dependent and -independent death pathways, through reducing the delayed accumulation of zinc in the vulnerable neurons in the hippocampus of the ischemic gerbil.

However, CQ could also act by alternative pathways involving modulation of cellular biometal metabolism. After administration of CQ, the normalization of zinc and copper reuptake in the glutamatergic synapse may improve the function of the NMDA receptor and restore long-term potentiation (LTP) [18]. The ionophoric activity of CQ has been shown to activate PI3K-Akt pathway through a mechanism that involves raising cellular metal levels [19], which might lead to neurons survival in the hippocampus of the ischemic gerbil, too. Moreover, CQ-zinc complex can cross the plasma membrane and intrcellular membranes, thereby decrease the free zinc concentration of cytoplasm by transport zinc to intracellular organelles such as mitochondria and lysosomes, which induced cancer cells apoptosis, but not normal cells [16]. Thus, there are other mechanisms of CQ involved in neuroprotective effects after ischemic insult.

In summary, we present data showing that administration of the zinc chelator, CQ, markedly reduces chelatable zinc accumulation, prevents neurodegeneration, and is accompanied by downregulation of zinc-triggered caspase-3, -9, and AIF activation in the gerbil hippocampus after ischemic insult. The present study indicates that the neuroprotective effect of CQ, likely involves modulation of both caspase-dependent and -independent apoptotic pathways.

## Materials and Methods

### Ethics statement

All efforts were made to minimize animal suffering and the number of animals used. The experimental procedures were carried out in accordance with the regulations of the animal protection laws of China and approved by the animal ethics committee of China Medical University (JYT-20060948).

### Animals and treatment

Adult male Mongolian gerbils weighing 60–80 g were used in this study. They were housed under a 12 h light/dark cycle with water and food available *ad libitum*.

The surgical procedure to produce transient global ischemia was carried out according to the method described previously with minor modification [36], [37]. Briefly, gerbils were anesthetized intraperitoneally (i.p.) with pentobarbital (40 mg/kg) and a 2.5 cm ventral neck incision was made. The bilateral common carotid arteries were separated carefully from the vagus nerves and were occluded bilaterally for 10 min with non-traumatic aneurysm clips. Then, the aneurysm clips were removed and complete reperfusion of the arteries was confirmed by direct visual observation. After suturing the neck incision area, gerbils were kept under a heating lamp for 2 h until they recovered. CQ (10 mg/kg, i.p., Sigma) or vehicle (DMSO, Sigma) was injected into the gerbils immediately after cerebral ischemia and then was given once a day untill the animals were sacrificed. The sham-operated non-ischemia gerbils underwent the same surgical procedures, except that the bilateral common carotid arteries were not occluded. All efforts were made to minimize the number of animals used and their suffering.

### TSQ fluorescence staining

TSQ (Molecular Probes, Eugene, OR) fluorescence dye staining was carried out as described previously [38], [39]. Briefly, three days after the production of transient global ischemia, gerbils (n = 6 in each group) were given the last injection of CQ or vehicle and, one hour later, the animals were sacrificed by giving an overdose of pentobarbital. The brains were removed immediately and cryosections (20 µm) were prepared. The sections were immersed in a solution of TSQ in 140 mM sodium barbital and 140 mM sodium acetate buffer (pH 10) for 90 s, followed by washing with normal saline for 60 s. TSQ fluorescence was observed under a fluorescence microscope with an ultraviolet filter.

### Zinc autometallography

The immersion AMG was performed as we described previously [40]. Three days after surgery, the gerbils (n = 6 in each group) received an injection of CQ or vehicle. One hour later, they were sacrificed and their brains were carefully removed. Fresh brain slices (2 mm) were prepared with a vibratome and immersed in NTS solution containing 0.1% sodium sulphide and 3% glutaraldehyde in 0.1 M phosphate buffer (PBS, pH 7.4) for 72 h at 4°C. After rinsing with PBS, the brain slices were immersed in 30% sucrose overnight at 4°C, and were then cut into 20 µm coronal sections in a cryostat. Five sections from each animal were selected and were incubated in the AMG developer in the same jar at 26°C for 60 min. The AMG developer was prepared as described previously [41]. After several rinses in distilled water, the sections were dehydrated, covered, and analyzed with a light microscope equipped with a digital camera. The DEDTC (Merck) control procedure was performed as reported previously [27], to confirm the specificity of the zinc staining.

### Nissl staining

At 1, 3 and 7 d post-surgery, the gerbils in each group (n = 6) were perfused transcardially with 4% paraformaldehyde in 0.1 M PBS. Their brains were then removed and embedded in paraffin according to standard protocols. When the intact morphology of the hippocampus was found in a section, it was considered as the beginning. Thus, seven series of paraffin sections (7 µm) were prepared and 5 series of sections were selected for Nissl, TUNEL, caspase-3, csapase-9, and HE staining, respectively.

For Nissl staining, sections were immersed in 0.1% cresyl violet at 37°C for 20 min. After rinsing with distilled water, sections were dehydrated, fitted with coverslips and examined with a light microscope. To assess neuronal survival in the CA1 region, neurons with round and palely stained nuclei were considered as surviving, while shrunken cells with pyknotic nuclei were considered as not surviving. Five brain sections were selected from each animal and processed for counting. Data were expressed as the number of surviving cells/field, as we reported previously [42].

### TUNEL staining

TUNEL staining of paraffin sections was performed according to the manufacturer's protocol (Roche, Germany). Briefly, sections of gerbil brain were incubated with 2% H_2_O_2_ in PBS for 5 min, and with a 20 µg/ml proteinase K working solution for 15 min at 37°C. After rinsing with 0.1 M PBS, sections were incubated in the TUNEL reaction mixture for 60 min at 37°C in a humidified atmosphere in the dark. The Converter-POD was added to the sections for 30 min at 37°C. After rinsing, sections were incubated with 0.025% 3,3′-diaminobenzidine plus 0.0033% H_2_O_2_ for 10 min. After rinsing, sections were counterstained with hematoxylin and examined with a light microscope. To assess TUNEL-positive cell expression, brown grains clustered over hematoxylin stained cells were counted and analyzed. Five brain sections were selected from each animal (n = 6) and cell counting was carried out. Data were expressed as the number of TUNEL-positive cells/field. As a negative control, the step using the TUNEL reaction mixture was omitted, and a nucleotide mixture in reaction buffer was used instead.

### *In situ* hybridization

In situ hybridization was carried out using a commercially available kit (Boster Biological Technology), with high-performance liquid chromatography (HPLC)-purified oligonucleotide probes specific for caspase-3 and caspase-9 mRNA. The mRNA expression was assessed on paraffin sections of brain at the level of the hippocampus from sham, vehicle- and CQ-treated ischemic gerbils at 3 d after the insult. Sections were acetylated, and incubated with pre-hybridization solution for 4 h at 40°C, and with the probes of caspase-3, and caspase-9 at 40°C, overnight. After rinsing, sections were treated with confining liquid at 37°C for 30 min. Then the ABC kit was applied for 1 h at room temperature, and a brown color appeared in the sections after incubation of the sections in 0.025% DAB with 0.0033% H_2_O_2_ for 10 min. Further processing of the stained sections was as described above. As a control, sections were incubated with a nonsense probe, which resulted in no detectable signal.

### Western blot analysis

Hippocampi isolated from gerbil brains were homogenized at a ratio of 1∶4 (w/v) in an ice-cold lysis buffer (50 mM Tris–HCl, 150 mM NaCl, 1% Nonidet P-40, 1 mM EDTA, 0.25% sodium deoxycolate, 0.1% SDS, 1 mM phenylmethylsulfonyl fluoride, 10 mg/ml leupeptin, 1 mM Na_3_VO_4_, and 1 mM NaF). The resulting homogenate was centrifuged at 12,000 g for 30 min at 4°C. The supernatant was collected and total protein levels were measured using a BCA protein assay kit (Pierce Biotechnology).

Sample protein (30 µg) was separated on 10% SDS-polyacrylamide gels and transferred onto PVDF membranes (Milipore). The membranes were blocked with 5% nonfat milk in TBS containing 0.05% Tween 20 for 2 h and then incubated with primary antibody for 2 h at room temperature. The dilutions of primary antibodies were 1∶500 for caspase-3 (Santa Cruz) and AIF (Santa Cruz), and 1∶12,000 for GAPDH (Kang Chen). Then, membranes were washed and incubated in HRP-conjugated goat anti-rabbit antibody (1∶5000) or HRP-conjugated goat anti-mouse antibody (1∶5000) for 1 h. Immunolabeled protein bands were detected using an enhanced chemiluminiscence system. Films were digitized using a scanner, and the relative optical density of the bands was determined with Image Scion software 4.03.

### Statistical analysis

All statistical analyses were carried out using an SPSS 13.0 software package. All values were represented as mean ± SD. Student's *t*-test was used for comparison between vehicle control and CQ treatment groups at each time point. *p*<0.05 was considered statistically significant.
